# *In vitro* transposition of ISY*100*, a bacterial insertion sequence belonging to the Tc*1*/*mariner* family

**DOI:** 10.1111/j.1365-2958.2007.05842.x

**Published:** 2007-09

**Authors:** Xiaofeng Feng, Sean D Colloms

**Affiliations:** Institute of Biomedical and Life Sciences, Division of Molecular Genetics, University of Glasgow, Anderson College 56 Dumbarton Rd, Glasgow G11 6NU, Scotland, UK

## Abstract

The *Synechocystis* sp. PCC6803 insertion sequence ISY*100* (IS*TcSa*) belongs to the Tc*1*/*mariner*/IS*630* family of transposable elements. ISY*100* transposase was purified and shown to promote transposition *in vitro*. Transposase binds specifically to ISY*100* terminal inverted repeat sequences via an N-terminal DNA-binding domain containing two helix–turn–helix motifs. Transposase is the only protein required for excision and integration of ISY*100*. Transposase made double-strand breaks on a supercoiled DNA molecule containing a mini-ISY*100* transposon, cleaving exactly at the transposon 3′ ends and two nucleotides inside the 5′ ends. Cleavage of short linear substrates containing a single transposon end was less precise. Transposase also catalysed strand transfer, covalently joining the transposon 3′ end to the target DNA. When a donor plasmid carrying a mini-ISY*100* was incubated with a target plasmid and transposase, the most common products were insertions of one transposon end into the target DNA, but insertions of both ends at a single target site could be recovered after transformation into *Escherichia coli*. Insertions were almost exclusively into TA dinucleotides, and the target TA was duplicated on insertion. Our results demonstrate that there are no fundamental differences between the transposition mechanisms of IS*630* family elements in bacteria and Tc*1*/*mariner* elements in higher eukaryotes.

## Introduction

Members of the Tc*1*/*mariner* family of transposable elements are widespread in nature, being found in eukaryotes ranging from insects and nematodes to fish and humans (reviewed in Plasterk *et al*., 1999). These elements have a simple structure, consisting of a single transposase gene flanked by terminal inverted repeats that mark the transposon ends. The transposase proteins have N-terminal DNA-binding domains that specifically recognize sequences within the transposon inverted repeats (Colloms *et al*., 1994; Vos and Plasterk, 1994; Wang *et al*., 1999; Izsvak *et al*., 2002), and C-terminal DDE or DDD domains that catalyse the transposition reactions (Doak *et al*., 1994; Vos and Plasterk, 1994; Richardson *et al*., 2006). Transposition is exclusively into TA target sequences, and insertions are flanked by duplications of the TA target.

Naturally occurring active elements from this family include *Mos1 mariner* from *Drosophila mauritiana* (Bryan *et al*., 1990) and Tc*1* and Tc*3* from *Caenorhabditis elegans* (Emmons *et al*., 1983; Collins *et al*., 1989). Other active elements, such as *Sleeping Beauty*, *Himar1* and *Frog Prince*, have been reconstructed from the sequences of multiple inactive copies present in the genomes of fish, insects and frogs respectively (Lampe *et al*., 1996; Ivics *et al*., 1997; Miskey *et al*., 2003). Transposons from the Tc*1*/*mariner* family are highly active in cultured cells and model organisms, and there is much interest in developing them for applications in biotechnology (reviewed in Ivics and Izsvak, 2004).

The Tc*1*/*mariner* family has been extensively studied, and *in vitro* transposition systems have been set up for a number of different elements (Lampe *et al*., 1996; Vos *et al*., 1996; Tosi and Beverley, 2000; Dawson and Finnegan, 2003). All of the major steps of transposition are catalysed by transposase, and no other host proteins are required. Transposition occurs by a cut and paste mechanism in which the transposon ends are first cleaved two to three nucleotides inside the transposon to produce 5′ phosphate termini, and exactly at the transposon ends on the opposite strands to produce 3′ hydroxyl termini (van Luenen *et al*., 1994; Lampe *et al*., 1996; Dawson and Finnegan, 2003). The 3′ hydroxyl groups at the transposon ends are transferred to 2 bp staggered positions on opposite strands at a TA target, and the strand transfer product is repaired by host machinery to produce the TA target duplication. Unlike Tn*5* and Tn*10*, double-strand cleavage at Tc*1*/*mariner* ends occurs without a hairpin intermediate (Dawson and Finnegan, 2003). The structure of the catalytic domain of *Mos1 mariner* from *D. mauritiana* has been determined by X-ray crystallography (Richardson *et al*., 2006). It has an RNase H-like fold similar to other DDE transposases, but lacks the hairpin binding pocket found in Tn*5* transposase (Davies *et al*., 2000).

**Fig. 1:**
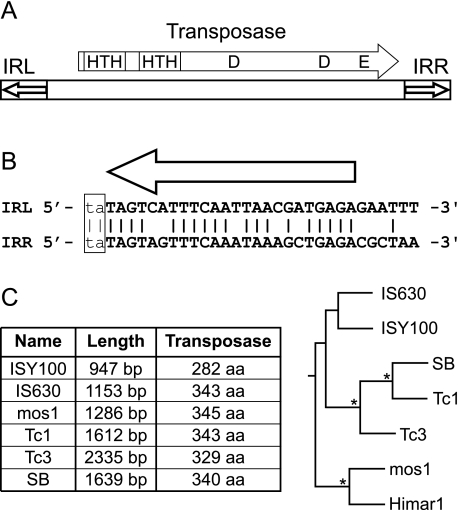
The structure of ISY*100*. A. ISY*100* encodes a single protein (transposase) flanked by two imperfect inverted repeats (IRL and IRR). Transposase contains two N-terminal helix–turn–helix DNA-binding motifs (HTH), and a C-terminal domain with homology to the DDE superfamily of DNA transposases. B. The sequences of IRL and IRR are shown aligned. Vertical lines show positions of identity and the flanking TA target duplication is shown lower case and boxed. The orientation of the inverted repeat sequences is indicated by an arrow above the sequences as in (A). C. Comparison of ISY*100* with other members of the Tc*1*/*mariner* family. Transposase lengths are shown in amino acids (aa). The phylogenetic tree was produced by PHYLIP NEIGHBOUR from a MUSCLE alignment of the C-terminal domains as shown in Fig. S2 (SB, *Sleeping Beauty*). Nodes supported by bootstrap values > 95% are indicated (*).

Members of the IS*630* family of Insertion Sequences are found in a wide range of bacterial species, from Proteobacteria such as *Escherichia coli* O157 to Cyanobacteria and Archaebacteria (Siguier *et al*., 2006). These elements appear to be distantly related to the Tc*1*/*mariner* family of transposons. They have a similar structure: they are flanked by apparent TA target duplications and carry single transposase genes whose products share sequence similarity with Tc*1*/*mariner* transposases (Fig. 1; Mahillon and Chandler, 1998). Two members of the IS*630* family, IS*630* from *Shigella sonnei* (Tenzen *et al*., 1990) and ISY*100* (otherwise known as IS*S1987* and IS*TcSa*) from *Synechocystis* sp. PCC6803 (Cassier-Chauvat *et al*., 1997; Urasaki *et al*., 2002), have been studied *in vivo*. Both elements transpose in *E. coli* upon expression of their cognate transposase protein. They are excised by double-strand breaks at the transposon ends and insert into TA dinucleotides with characteristic two base-pair target duplications (Tenzen *et al*., 1990; Tenzen and Ohtsubo, 1991; Urasaki *et al*., 2002). However, to date no bacterial elements from this family have been studied *in vitro*. To study the mechanism of transposition of these bacterial elements in more detail, and to allow comparison with their eukaryotic counterparts, we have set up an *in vitro* transposition system for ISY*100*. Here we report that transposase is the sole protein required for transposition and demonstrate that transposition occurs by a cut and paste mechanism similar to that of other characterized members of the Tc*1*/*mariner* family. Our results demonstrate the evolutionary conservation of transposition mechanism of Tc*1*/*mariner*/IS*630* elements, and provide a genetically and biochemically tractable system for the study of this family.

## Results

### Purification of ISY*100* transposase

ISY*100* is 947 bp in length, contains 24 bp imperfect inverted repeats (IRL and IRR) and encodes a single transposase protein of 282 amino acids (Fig. 1). The transposase gene from an active copy of ISY*100* was isolated by PCR, and placed together with DNA sequences encoding a C-terminal 6-His tag under the control of the strong T7 promoter in the expression plasmid pKET3a. Control experiments demonstrated that this His-tagged transposase was as active as the wild-type transposase in an *in vivo* transposition assay in *E. coli* (data not shown). Transposase was expressed in *E. coli* and purified using nickel affinity chromatography as described in *Experimental procedures*. Transposase was soluble in a buffer containing 1 M NaCl and 0.1% Triton X-100, and was essentially pure as judged by Coomassie-stained SDS-polyacrylamide gel electrophoresis (Fig. S1).

### Transposase binds to ISY*100* inverted repeat sequences

**Fig. 2:**
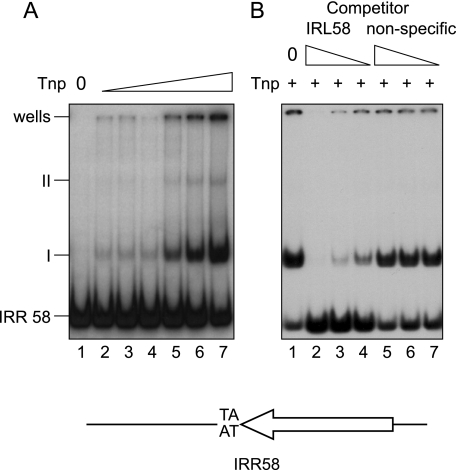
Binding of full-length transposase to ISY*100* IRR. A. A 58 bp DNA fragment containing 30 bp from the right end of ISY*100* and 28 bp of flanking sequences (IRR58) was incubated with purified transposase, and complexes were separated by non-denaturing polyacrylamide gel electrophoresis. Unbound DNA (IRR58) and two complexes (I and II) are indicated. A twofold dilution series of transposase was used: Lane 1, no protein; lane 2, ∼3 nM transposase; lane 7, ∼100 nM transposase. B. Competition experiment. All lanes contain labelled IRR58 and 100 nM transposase. Lane 2, 3 and 4 contain 1 μM, 0.33 μM and 0.1 μM, respectively, of unlabelled IRL58 as competitor, whereas lanes 5, 6 and 7 contain the same concentrations of an unrelated 53 bp sequence.

To test whether transposase can bind to DNA, an electrophoretic mobility shift assay (EMSA) was carried out with purified transposase and a 58 bp labelled DNA fragment (IRR58) containing 30 bp from the right end of ISY*100* together with 28 bp of flanking sequences. Transposase bound to IRR58 in the presence of an excess of poly(dI-dC) competitor, giving one major retarded complex (complex I, Fig. 2A). Transposase also bound to ISY*100* IRL with a similar affinity (data not shown). Binding was abolished by the addition of excess unlabelled IRL58 competitor DNA, but not by the addition of an equivalent amount of unrelated DNA (Fig. 2B). Therefore the binding is specific for transposon inverted repeat sequences. On some gels, a minor slower migrating complex was observed (complex II; Fig. 2A). Addition of an excess of a larger (78 bp) unlabelled fragment containing IRL sequences (IRL78) reduced the mobility of the labelled complex II formed by IRR58, but had no effect on complex I (data not shown), demonstrating that complex I contains only one DNA fragment, while complex II contains at least two DNA fragments and may represent a paired end complex (PEC).

**Fig. 3:**
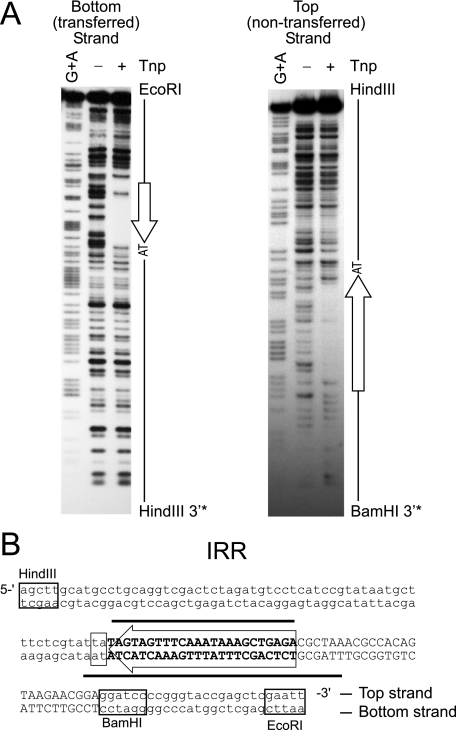
DNase I footprint of full-length transposase bound to IRR. A. Transposase protects the inverted repeat sequence (shown as an arrow) on both strands of IRR. B. DNA sequence of the restriction fragment used in footprinting experiments. The 49 bp derived from the right end of ISY*100* are shown in capital letters and the 24 bp inverted repeat is indicated with an arrow. The extent of protection from DNase I on top and bottom strands is indicated by black bars above and below the sequence respectively.

To ascertain where transposase binds on the transposon ends, DNase I footprinting experiments were carried out on DNA fragments containing ISY*100* IRR (Fig. 3A). Almost all of the 24 bp IRR sequence was protected on the top strand, while the complete IRR sequence and a few nucleotides on either side were protected on the bottom strand (Fig. 3B). A similar pattern of protection was observed on DNA fragments containing IRL (data not shown).

### ISY*100* transposase contains an N-terminal DNA-binding domain

**Fig. 4:**
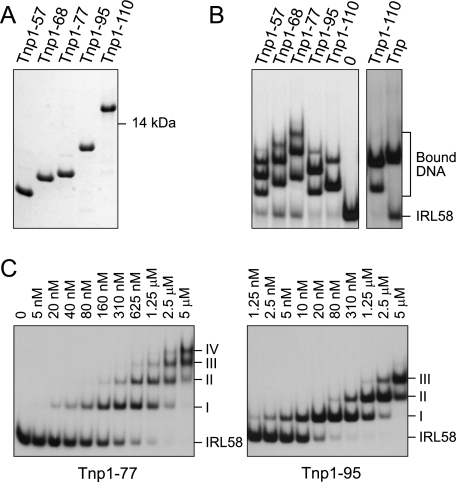
Binding of N-terminal transposase derivatives to the ISY*100* inverted repeat. A. Transposase deletion derivatives were purified by metal affinity chromatography and analysed by Coomassie-stained Tris-tricine SDS-PAGE. B. The indicated protein (1.25 μM) was incubated with IRL58, and protein–DNA complexes were separated by non-denaturing polyacrylamide gel electrophoresis. C. The indicated concentrations of Tnp_1−77_ and Tnp_1−95_ were incubated with IRL58 and complexes were separated as in (B).

N-terminal DNA-binding domains that specifically recognize their transposon terminal sequences have been identified in a number of Tc*1*/*mariner* family transposases (Colloms *et al*., 1994; Vos and Plasterk, 1994; Auge-Gouillou *et al*., 2001; Zhang *et al*., 2001; Izsvak *et al*., 2002; Watkins *et al*., 2004; Feschotte *et al*., 2005). To test whether ISY*100* transposase contains a similar DNA-binding domain, N-terminal derivatives containing the first 37, 38, 46, 57, 68, 77, 95 or 110 amino acids of ISY*100* transposase (Tnp_1−37_ … Tnp_1−110_) were expressed in *E. coli* and tested for their ability to bind to DNA. Crude extracts from *E. coli* expressing Tnp_1−37_, Tnp_1−38_ and Tnp_1−45_ failed to bind to ISY*100* inverted repeat sequences, while crude extracts containing Tnp_1−57_, Tnp_1−68_, Tnp_1−77_, Tnp_1−95_ and Tnp_1−110_ did bind (data not shown). To investigate this further, Tnp_1−57_, Tnp_1−68_, Tnp_1−77_, Tnp_1−95_ and Tnp_1−110_ were purified by nickel affinity chromatography (Fig. 4A) and used in EMSAs with IRL58. Tnp_1−57_, Tnp_1−68_, Tnp_1−77_ bound to IRL58, giving a series of three to four retarded complexes that decreased in mobility with increasing protein length (Fig. 4B). Tnp_1−95_ and Tnp_1−110_ also produced a series of retarded complexes with IRL58, but there was a step shift in behaviour between Tnp_1−77_ and Tnp_1−95_ (Fig. 4B). The fastest-migrating complexes produced by Tnp_1−95_ and Tnp_1−110_ had higher mobilities than the fastest complex produced by Tnp_1−77_. To see if this step shift was reflected in the binding affinity of these two proteins, dilutions of Tnp_1−77_ and Tnp_1−95_ were used in EMSAs with IRL58 (Fig. 4C). Both proteins gave a series of retarded bands, with the slower-migrating complexes appearing only as the protein concentration was increased above approximately 100 nM. Experiments with mixtures of different-length DNA fragments gave no indication that any of these complexes contained more than one DNA fragment (data not shown). The slower-migrating complexes most likely result from binding of multiple protein subunits to IRL58, either by non-specific DNA binding or by protein–protein interactions with specifically bound subunits. Tnp_1−95_ bound to IRL58 about 16-fold more tightly than Tnp_1−77_, with apparent disassociation constants (*K*_d_) for the first complexes of approximately 10 nM and 160 nM respectively. This difference in affinity was also reflected in DNase I footprinting experiments on transposon ends. Tnp_1−95_ gave almost identical protection to the full-length transposase, whereas no specific protection was observed with Tnp_1−57_ and Tnp_1−77_ (data not shown).

Tc*3* transposase contains two helix–turn–helix DNA-binding domains, that bind Tc*3* terminal inverted repeat sequences in the major groove and are separated by an ‘AT hook-like’ basic sequence that binds in the minor groove (Watkins *et al*., 2004). To see if ISY*100* transposase contains similar DNA-binding domains, the program *helixturnhelix* (Dodd and Egan, 1990; Rice *et al*., 2000) was used to search for helix–turn–helix motifs in ISY*100* transposase. Two helix–turn–helix motifs were found in the N-terminal domain of ISY*100* transposase, between residues 19–40 and 71–92 (Fig. S2A). A multiple sequence alignment-based secondary structure analysis (jpred; Cuff and Barton, 2000) predicted six α-helices in the N-terminal domain of transposase, with helices 2–3 and 5–6 making up the two helix–turn–helix motifs. A basic region with homology to the AT hook-like motif in Tc*3* transposase is also present in ISY100 transposase, between predicted α-helices 3 and 4. Furthermore, a hidden Markov model-based homology search detected convincing sequence similarity between the N-terminal domain of ISY*100* transposase and the equivalent region of Tc*3* transposase and other paired-like DNA-binding domains. It therefore seems likely that ISY*100* transposase contains two helix–turn–helix DNA-binding domains separated by an AT hook-like structure that binds to DNA just like the N-terminal domain of Tc*3* transposase.

Our binding data are nicely explained by the predicted structure of the N-terminal DNA-binding domain. Tnp_1−45_ and smaller derivatives lack the AT hook region and/or part of the first helix–turn–helix and fail to bind to DNA. Tnp_1−57_, Tnp_1−68_ and Tnp_1−77_ contain only the first helix–turn–helix and AT hook, and therefore bind relatively weakly to the transposon end, whereas Tnp_1−95_ and Tnp_1−110_ contain both helix–turn–helices, and bind more tightly. The structure may also explain the relative gel mobilities of complexes formed by these proteins. The incomplete C-terminal helix–turn–helix in Tnp_1−77_ is unlikely to bind to DNA, and may retard the protein–DNA complexes compared with those formed by Tnp_1−95_ and Tnp_1−110_.

### ISY*100* transposase cleaves transposon ends

**Fig. 5:**
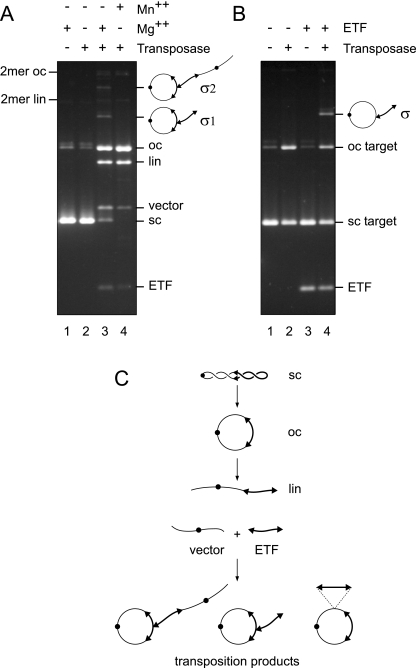
*In vitro* excision and transposition of ISY*100*. A. Supercoiled pISY*100*-kan (4.0 kb), containing a 1.3 kb kanamycin resistance fragment flanked by IRL and IRR in pUC18, was incubated for 16 h in the presence of transposase (100 nM) and divalent cations as indicated. Products were separated by agarose gel electrophoresis. Bands are indicated as follows: sc, lin and oc, supercoiled, linear and open circle pISY*100*-kan, respectively; 2mer lin and 2mer oc, linear and open circle dimer; ETF, excised mini-transposon fragment; vector, vector backbone. Proposed intermolecular transposition products (σ1 and σ2) are indicated schematically. B. Supercoiled pUC4K target plasmid (3.9 kb) was incubated with or without purified excised mini-transposon fragment and transposase as indicated. Bands are indicated as in (A) except for supercoiled and open circle pUC4K, sc target and oc target. C. Proposed transposition pathway. Single-strand cleavage at one or both transposon ends produces open circle. Cleavage of the other strand at one or both ends releases ETF, vector and linearized substrate. Linear and ETF fragments can transpose into another copy of the circular substrate to produce the intermolecular transposition products shown.

To test whether ISY*100* transposase has catalytic activity, a plasmid (pISY*100*-kan) containing an artificial mini-transposon, consisting of IRL and IRR flanking a kanamycin-resistance gene, was incubated in a simple buffer with purified full-length transposase (Fig. 5A). Supercoiled pISY*100*-kan was converted to a number of different products in a reaction that was dependent on transposase and either Mg^2+^ or Mn^2+^ divalent cations. The reaction produced linearized plasmid and two fragments of the correct size to be excised mini-transposon (ETF) and the vector backbone. Restriction digestion confirmed that these products were produced by double-strand cleavage at IRR and IRL. Most of the linearized plasmid was produced by cleavage at IRL (∼80%) while the rest (∼20%) was produced by cleavage at IRR (data not shown). A significant amount of nicked (open circle) plasmid was also produced by transposase in these reactions (Fig. 5A, lanes 3 and 4). It seems likely that transposase has cleaved one strand at one or both transposon ends in these open circular products. Nicked and linear dimer, produced from circular dimer present in the substrate, were also observed (Fig. 5A, lanes 3 and 4).

To test whether supercoiling is required for cleavage by transposase, linearized pISY*100*-kan was used in a cleavage assay. Transposase cleaved linearized pISY*100*-kan poorly under standard conditions, but the requirement for supercoiling could be overcome, at least in part, by the addition of 10–20% dimethyl sulphoxide (DMSO) to the reaction buffer (data not shown), as has been shown in other *in vitro* transposition systems (e.g. Mizuuchi and Mizuuchi, 1989).

Two prominent bands migrating between open circle monomer and open circle dimer were produced from pISY*100*-kan only in the presence of Mg^2+^ (σ1 and σ2; Fig. 5A, lane 3). To see if these bands might be the result of intermolecular transposition, reactions were carried out using gel-purified excised mini-transposon and circular target plasmids of different sizes. A major product, running slower than the open circle form of the target plasmid, was observed with all targets (σ; Fig. 5B, lane 4, and data not shown). Restriction analysis revealed that just one end of the linear transposon fragment had inserted into the target plasmid in these products (data not shown). When the target plasmid was approximately the same size as pISY*100*-kan, single-end insertion products had a similar mobility to one of the products observed with pISY*100*-kan alone (σ1 in Fig. 5A). It therefore seems likely that σ1 consists of a mini-transposon excised from ISY*100*-kan, and inserted into a second circular copy of the substrate plasmid. The slower product seen in Fig. 5B (σ2) could consist of a full-length copy of the substrate plasmid, linearized at one transposon end and inserted into another circular copy of the substrate (Fig. 5C).

### Mapping the cleavage sites on ISY*100* ends

**Fig. 6:**
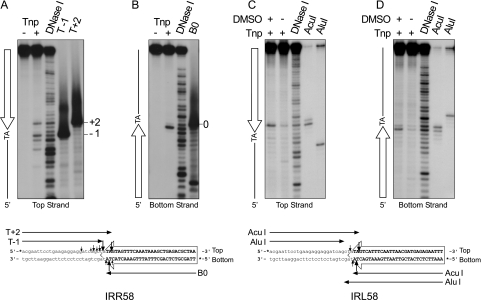
Mapping the cleavage sites on ISY*100* ends. A. IRL58 was 5′ end-labelled with ^32^P on the top strand and incubated in the presence of 20% DMSO with or without transposase as indicated. Reactions were run on an 8% polyacrylamide sequencing gel, alongside a marker ladder produced by limited DNase I cleavage of the same DNA. Labelled single-stranded oligonucleotides (T+2 and T−1), representing predicted cleavage products, were run as additional markers. B. IRL58 was 5′ end-labelled with ^32^P on the bottom strand and treated as in A. Labelled single-stranded oligonucleotide representing the predicted cleavage product (B0) was run as marker. C. IRR58 was 5′ end-labelled with ^32^P on the top strand and incubated with transposase in the presence or absence of 20% DMSO as indicated. Reactions were run on an 8% polyacrylamide sequencing gel adjacent to a DNase I ladder produced from the same labelled DNA. Additional markers were produced by cleavage with AcuI or AluI. D. IRR58 was 5′ end-labelled with ^32^P on the bottom strand and treated as in C. Major and minor transposase cleavage sites (large and small arrows), and marker sizes are indicated on the DNA sequence below the gels.

To map the exact sites of cleavage at ISY*100* ends, double-stranded oligonucleotides containing IRR or IRL sequences were 5′ end-labelled on the top or bottom strands, and incubated with transposase in the presence of Mg^2+^ and DMSO. Reaction products were run on a strand-separating polyacrylamide gel adjacent to appropriate single-stranded markers. Top strand cleavage (yielding a transposon 5′ end) occurred at several positions close to the transposon end. A strong cut site was present two nucleotides inside the transposon on both IRR and IRL, but cleavage also occurred one to eight nucleotides outside the transposon at both ends (Fig. 6A and C). On the bottom strand, the majority of cleavage was one nucleotide inside the transposon, although a small amount of cleavage was detected precisely at the transposon 3′ end (Fig. 6B and D).

The finding that transposase cleaves one nucleotide inside the transposon at the 3′ end is highly surprising. All DDE transposases are thought to cleave precisely at the transposon 3′ end. Furthermore, Urasaki *et al*. (2002) reported that ISY*100* is cleaved precisely at the transposon 3′ end *in vivo*, and cleavage inside the transposon on the transferred strand will lead to loss of transposon sequences during transposition. To test whether the position of cleavage was altered by the presence of DMSO in the reaction buffer, reactions were carried out in the absence of DMSO. In these conditions, cleavage occurred with reduced efficiency, but the specificity of cleavage did not appear to be greatly altered (Fig 6C and D, compare lanes 1 and 2).

**Fig. 7:**
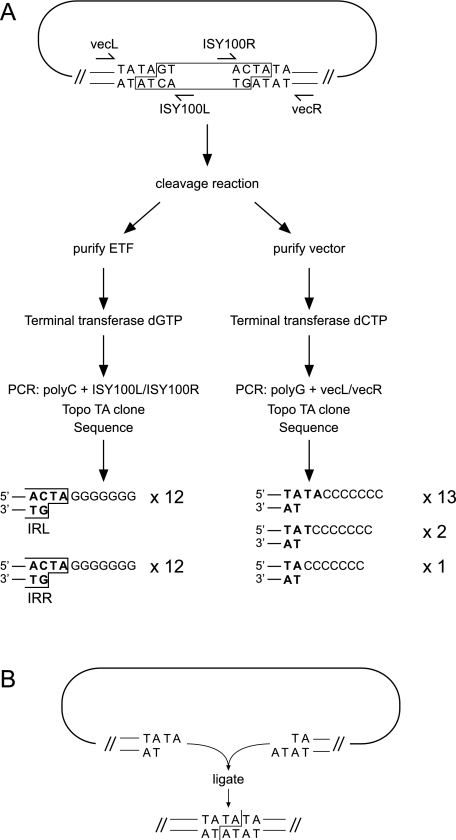
Mapping cleavage positions on supercoiled substrates. A. Transposase was incubated with supercoiled plasmids containing ISY*100* or ISY*100*-kan to give ETF and vector fragments, which were purified from an agarose gel. 3′ OH ends were tailed using terminal transferase and dCTP or dGTP. Tailed products were amplified by PCR using the indicated primers and cloned by topo-TA cloning. The end-tail junction sequences obtained and their frequencies are shown. B. The vector fragment was circularized with T4 DNA ligase, giving the junction sequence shown.

To see if cleavage one nucleotide inside the transposon end occurs only on small linear substrates, transposase cleavage sites were mapped on plasmid substrates containing mini-transposons. After *in vitro* cleavage with transposase, the ETF and the vector backbone fragments were purified by gel electrophoresis and G- or C-tailed using terminal transferase. End-tail junctions were amplified by PCR with poly C or poly G primers together with vector- or transposon-specific primers (Fig. 7A). PCR products were ligated into a cloning vector and isolated by transformation into *E. coli.* Individual end-tail junctions were then determined by DNA sequencing. This analysis indicated that cleavage on plasmid substrates occurred exactly at the transposon 3′ ends (24 out of 24 IRR and IRL G-tail junctions; Fig. 7A) and almost always two nucleotides inside the transposon 5′ ends (13 out of 16 vector C-tail junctions; Fig. 7A). Consistent with this result, gel-purified vector backbone produced by transposase-mediated cleavage of pISY*100*-kan was efficiently circularized with T4 DNA ligase and could be recovered after transformation into *E. coli*, yielding plasmids with the predicted TATATA junction sequence (Fig. 7B).

Terminal transferase requires the presence of 3′ hydroxyl ends, and T4 DNA ligase requires 3′ OH and 5′ phosphate termini. These results, together with the migration of cleavage products relative to the DNase I ladder in Fig. 6, therefore indicate that transposase cleaves to produce 3′ OH and 5′ phosphate ends on both strands.

### Transposon 3′ ends are transferred to target DNA

**Fig. 8:**
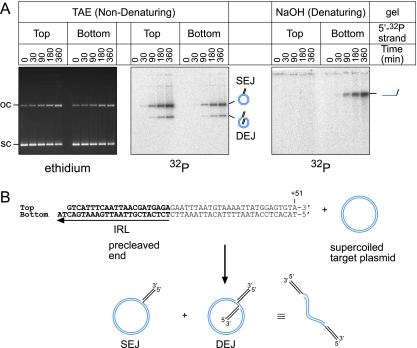
The 3′ end of ISY*100* becomes covalently linked to the target DNA in the strand transfer reaction. A. A pre-cleaved transposon end, 5′ end-labelled on either the top or bottom strand, was incubated with transposase and a supercoiled target plasmid. Samples were withdrawn at the indicated times and run on a non-denaturing agarose gel (TAE) or a strand-separating alkaline gel (NaOH). DNA was detected by ethidium staining, and by autoradiography (^32^P). The positions of supercoiled (sc) and open circle (oc) target plasmid are shown to the left of the ethidium-stained gel. The positions of single end-joined (SEJ) and double end-joined (DEJ) strand transfer products are shown to the right of the autoradiographs. SEJ and DEJ products are indistinguishable on the strand separating gel. Unreacted labelled substrate has been cropped from the bottom of both autoradiographs. B. Sequence of the pre-cleaved end and schematic diagram of the reaction products.

To investigate the strand transfer activity of transposase, we used double-stranded oligonucleotides representing cleaved transposon ends. The termini were chosen to be the same as those produced on supercoiled plasmid substrates, exactly at the transposon 3′ end and two nucleotides inside the transposon at the 5′ end. Pre-cleaved transposon ends were labelled on either the top or bottom strand, and incubated together with a supercoiled target plasmid and transposase in a time course experiment. The products were run on a non-denaturing agarose gel and detected by autoradiography. Radioactive products co-migrating with the open circular and linear forms of the target plasmid were produced only in the presence of transposase, divalent metal ions and target plasmid (Fig. 8A and data not shown). The mobilities of these products are consistent with transfer of one or two transposon ends to a target plasmid, yielding nicked circle with one attached transposon end, and a linear molecule with two attached transposon ends respectively (Fig. 8B). Strand transfer products accumulated over time, and were observed irrespective of whether the top or the bottom strand was labelled (Fig. 8A). When the same reactions were run on a denaturing agarose gel, plasmid-sized labelled products were observed only when the transposon end was labelled on the bottom strand (Fig. 8A). Thus only the bottom strand, containing the transposon 3′ end, becomes covalently linked to the target plasmid in the strand transfer reaction.

Substrates mimicking aberrantly cut ISY*100* ends were also tested for strand transfer. These substrates ended one nucleotide inside the transposon on the bottom (transferred) strand, or one nucleotide outside the transposon on the top (non-transferred) strand. No strand transfer was observed when the bottom strand ended one nucleotide inside the transposon, and the efficiency of strand transfer was much reduced when the top strand was one nucleotide longer than the transposon (data not shown). It therefore appears that the main cleavage products observed in the reactions of linear substrates (Fig. 6) are dead ends in the reaction pathway.

### ISY*100* Transposase can catalyse a complete transposition reaction

To test whether purified transposase can catalyse a complete transposition reaction, we used a mini-transposon conferring resistance to kanamycin (ISY*100*-kan) carried on a plasmid with a λ origin of replication (pλISY*100*-kan). The supercoiled donor plasmid was mixed with a target plasmid conferring resistance to ampicillin, and incubated with transposase. Reaction products were electroporated into a λ lysogen *recA*^–^*E. coli* strain and transformants were selected for resistance to kanamycin. As the λ-dv donor plasmid cannot replicate in the λ lysogen strain, no kanamycin-resistant colonies were obtained in the absence of transposase. In the presence of transposase, kanamycin-resistant colonies were obtained with a frequency of 1.4–1.9 per 10^3^ ampicillin-resistant colonies (Table 1), indicating that approximately 1 in 500 target plasmids had received a copy of ISY*100*-kan. Restriction analysis of the resulting plasmids showed that they contained ISY*100*-kan insertions at different sites in the target plasmid. Sequencing 10 such transposition products demonstrated that they all contained the intact mini-transposon flanked by TA target duplications.

**Table 1:** Transposition frequencies measured by transformation into *E. coli.*

Donor^a^	Amp^r^Colonies^b^	Kan^r^Colonies^b^	Transposition Frequency^c^
pλISY*100*-kan	1.2 × 10^6^	1700	1.4 × 10^−3^
	1.3 × 10^6^	2530	1.9 × 10^−3^
Purified ETF	2.8 × 10^6^	3440	1.2 × 10^−3^

a pλISY*100*-kan, or purified excised ISY*100*::kan, was incubated with the supercoiled amp^r^ target plasmid pH2. Reactions were stopped with phenol and DNA was recovered by ethanol precipitation.

b Number of colonies obtained after one twentieth of the reaction was electroporated into the λ-lysogen strain DS964.

c Transposition frequency, Total number of Kan^r^ colonies/total number of Amp^r^ colonies. The first two lines of the table show two independent repeats of the same experiment.

Similar experiments were also carried out using excised transposon, released from pISY*100*-kan with the restriction enzyme AcuI, which cleaves at the exact same bonds as transposase. Gel-purified ISY*100*-kan was mixed with a target plasmid conferring resistance to ampicillin, incubated with transposase and the reactions were electroporated into *E. coli*, yielding kanamycin-resistant colonies at a frequency of approximately 1 per 1000 ampicillin resistant colonies in a transposase dependent reaction (Table 1). Restriction mapping of the resulting plasmid DNA indicated that they contained insertions of ISY*100*-kan at different sites in the target plasmid, and this was confirmed by DNA sequencing. Out of 39 transposition products analysed, 34 contained insertions into TA dinucleotides and had a normal TA duplication. Of the remaining five, two were inserted into and had duplicated AA/TT dinucleotides, and three had inserted into TA and were flanked by TA on one side but only A on the other.

## Discussion

We have purified the transposase from a bacterial IS*630*/Tc1/*mariner* family transposase and shown that it binds to transposon ends, where it carries out cleavage and strand transfer reactions. The TAs flanking all copies of ISY*100* are not part of the excised element and thus must be target duplications. Our results are fully consistent with the idea that transposition occurs by a cut and paste mechanism in which the transposon is excised by double-strand cleavages at both ends and then inserted into target sites by attack of 3′ hydroxyl ends at staggered positions at TA dinucleotides. Our results show that transposition of this bacterial IS*630* family element occurs by an identical mechanism to its Tc1/*mariner* counterparts, and that there are no fundamental differences between transposition in eukaryotes and prokaryotes.

In a 16 h reaction on a supercoiled substrate, almost all the substrate DNA was nicked, while only about 20% of the DNA had double-strand cleavages at one or both ends. *Mos1 mariner* transposase cleaves the two strands at transposon ends in a stepwise fashion, with the non-transferred strand cleaved before the transferred strand (Dawson and Finnegan, 2003), and results with Tc*1* are suggestive of a similar stepwise cleavage mechanism (Vos *et al*., 1996). The large amount of nicked substrate produced by ISY*100* transposase may reflect a similar sequential strand cleavage mechanism.

Strand transfer products were easily detected on ethidium-stained gels when a pre-cut excised mini-transposon was mixed with a supercoiled target DNA. The majority of these products contained insertions of only one transposon end into the target. Products with similar mobility were also observed in reactions on supercoiled plasmids containing mini-transposons, and it seems likely that these correspond to insertions of one end of the excised transposon into the substrate plasmid (Fig. 5C).

Although less frequent than single-end insertion events, genuine transposition events involving both transposon ends could be detected after electroporation into *E. coli*, and large numbers of transposition products could be isolated (> 40 000 per 20 μl of transposition reaction). At this stage, we cannot rule out the possibility that host factors are required to increase the efficiency or specificity of the transposition reaction. Nevertheless, the level of excision and transposition compares favourably with that observed in other *in vitro* transposition systems. Transposition occurs at a level that can be easily studied *in vitro*, and could be used for the generation of insertion mutant libraries or for other applications.

It has been reported that efficient transposition of other members of the Tc*1*/*mariner* family requires transposon internal sequences (Izsvak *et al*., 2002). However, we have observed no differences in efficiency of cleavage or transposition between plasmids carrying the full-length ISY*100* and those carrying mini-transposons with only 30 bp from each transposon end. The terminal inverted repeats of ISY*100* are 24 bp in length, and this corresponds well with the region protected in footprinting experiments. It therefore seems likely that the minimum *cis* requirements for ISY*100* transposition will be the 24 bp imperfect inverted repeats.

ISY*100* transposase is smaller than typical eukaryotic members of the Tc*1*/*mariner* family, and lacks approximately 30 amino acids corresponding to the extreme C-terminus of *Mos1* transposase. Despite its small size, ISY*100* transposase is highly active *in vitro* and *in vivo*. Other transposons from this family have been used to generate stable insertions of transgenic DNA in a wide variety of model organisms (Plasterk *et al*., 1999). Purified ISY*100* transposase can carry out all of the steps of a transposition reaction, and at most 30 bp of transposon sequences are required from each transposon end. We now have an *in vitro* transposition system that allows us to study the separate steps in the transposition reaction and we are currently developing genetic tools in *E. coli* for the selection of transposase variants with novel properties. For these reasons, ISY*100* transposase holds promise as a biotechnological tool to rival other members of this transposon family, especially if it can be shown to be active in mammalian as well as bacterial cells.

## Experimental procedures

### Overexpression of ISY*100* transposase

A known active copy of ISY*100*, located between 52202 and 53216 bp on the *Synechocystis* sp. PCC6803 genome (Kaneko *et al*., 1996), was isolated by PCR with Pfu polymerase using primers 5′-GGGGAATTCATATGTCCGGACTTCGCTATAGTTTCTAAAG and 5′-CCCGGATCCATGGTCTAGATGTCCTCATCCGTATAATGC, and inserted between the XbaI and EcoRI sites of pUC18 to create pXF106. PCR with primers 5′-GGAATTCCATATGGCTTACAGTTTAGA and 5′-CCCGGATCCCTAGTGGTGGTGGTGGTGGTGAACGCCACAGTAAGAACGGAT was then used to amplify the transposase gene and add a C-terminal 6-His tag. The His-tagged transposase gene was inserted into a kanamycin-resistant version of the pET3a expression vector (pKET3a) to make pXF102. The sequence of the transposase gene in pXF102 was verified by sequencing. An overnight culture of BL21 (DE3) pLysS (Studier *et al*., 1990) pXF102 was diluted 1:100 in fresh LB broth containing 50 μg ml^−1^ kanamycin and 25 μg ml^−1^ chloramphenicol, and grown with vigorous shaking at 37°C to an OD_600_ of 0.5. Transposase expression was induced by the addition of Isopropyl-β-d-thiogalactopyranoside to 0.5 mM. Cells were grown for a further 2 h at 37°C and harvested by centrifugation.

### Purification of transposase

All steps were carried out on ice or at 4°C. One gram of frozen cell pellet was re-suspended in 10 ml of TBP [50 mM Tris-HCl pH 7.5, 14.3 mM β-mercapto-ethanol, 1 mM phenylmethyl-sulphonylfluoride (PMSF)] and sonicated. An equal volume of TB (same as TBP but without PMSF) was added and the suspension was made up to 10 mM MgCl_2_. After centrifugation at 28 300 *g* for 15 min, 200 mM spermine was added slowly to the supernatant to a final concentration of 2.5 mM while stirring. After a further 10 min of stirring, the suspension was centrifuged at 16 000 *g* for 5 min, the pellet was re-suspended in 20 ml of buffer TBST (buffer TB with 1 M NaCl and 0.2% Triton X-100), and the mixture was stored on ice for 1 h. After centrifugation at 16 000 *g* for 15 min, the supernatant containing soluble transposase was applied to a Ni-NTA column (Qiagen) equilibrated with TBST. The column was washed with 10 column volumes of TBST followed by 6 column volumes of TBST plus 50 mM imidazole, and transposase was eluted in TBST plus 200 mM imidazole. Peak fractions were pooled and dialysed overnight against a buffer containing 50 mM Tris-HCl pH 7.5, 1 M NaCl, 0.1% Triton X-100 and 0.1 mM DTT. The dialysed protein was made up to 50% glycerol and stored at −20°C or −70°C.

### N-terminal transposase derivatives

Deletion derivatives of pXF102, encoding N-terminal fragments of transposase linked to a 6-His tag, were constructed using PCR with appropriate primers. Proteins were expressed in BL21 (DE3) pLysS as for full-length transposase. Cell pellets were re-suspended in 5 ml of 50 mM Tris-HCl pH 7.5, 0.5 M NaCl and 1 mM PMSF, sonicated and centrifuged at 28 300 *g* for 20 min. The supernatant was made up to 1 M NaCl, 0.2% Triton X-100, 10 mM imidazole and loaded on a His-gravitrap column (GE Healthcare). The column was washed with the same buffer containing 30 mM imidazole, and protein was then eluted with 200 mM imidazole.

### Electrophoretic mobility shift assays

Ten microlitres of binding reactions contained 0.04 pmoles of IRR58 (5′-acgaattcctgaagaggaggatcagctaTAGTAGTTTCAAATAAAGCTGAGACGCTAA, end-labelled with [γ-^32^P]-ATP and T4 polynucleotide kinase and annealed to its unlabelled complement) or the equivalent IRL58 fragment, and 0.5 μg of poly(dI-dC) in 10 mM Tris-HCl pH 7.5, 0.1 mg ml^−1^ bovine serum albumin (BSA), 0.1 mM EDTA, 1 mM dithiothreitol (DTT) and 10% glycerol. Binding was started by addition of 1 μl of transposase (or deletion derivative) diluted in protein dilution buffer (50% glycerol, 50 mM Tris-HCl pH 7.5, 1 M NaCl, 0.1% Triton X-100 and 0.1 mM DTT). Binding reactions were incubated at 30°C for 10 min and then run on 6% polyacrylamide gels in 1/3× TBE running buffer (30 mM Tris base, 30 mM boric acid and 0.33 mM EDTA) at 4°C.

### DNase I footprinting

pXF121, containing 49 bp of ISY*100* IRR in pUC18, was cleaved with BamHI or HindIII, 3′ end-labelled with [α-^32^P]-dATP and Klenow polymerase, and then cleaved with either EcoRI or HindIII. The resulting IRR49 fragment, was gel-purified, and 0.8 pmoles was used in a standard binding assay as described above. Cleavage was initiated by the addition of 1 μl of a 4 μg ml^−1^ solution of DNase I in 10 mM Tris-HCl pH 7.5, 50 mM CaCl_2_, 100 mM MgCl_2_. After 1 min at 20°C, cleavage was stopped by the addition of sequencing loading dye (95% formamide, 30 mM EDTA, 0.1% bromophenol blue and 0.1% xylene cyanol FF). Reactions were run on 8% polyacrylamide sequencing gels containing 7.5 M urea and 1× TTE (89 mM Tris base, 29 mM taurine and 0.5 mM EDTA).

### *In vitro* transposition assays using supercoiled donor and target plasmids

Donor plasmids contained IRL58 and IRR58 flanking the kanamycin resistance determinant from pUC4K (Vieira and Messing, 1982) in the polylinker of pUC18 or pCLIP18 (Boyd and Sherratt, 1995), giving pISY*100*-kan and pλISY*100*-kan respectively. ETF was obtained by AcuI digestion of pISY*100*-kan and purified from agarose gels. pUC4K and pH2, containing a 2639 bp TA-rich PstI–HindIII AMA1 fragment (Aleksenko and Clutterbuck, 1996) from *Aspergillus nidulans* in the cloning vector pIC20R, were used as target plasmids. Standard *in vitro* transposition assays contained 0.12 pmol of supercoiled donor plasmid or 0.30 pmol of purified ETF, together with 0.3 pmol of pH2 or 0.12 pmol of pUC4K in 20 μl of transposase assay buffer (10 mM MgCl_2_, 1 mM DTT, 0.1 mg ml^−1^ BSA, 50 mM Tris-HCl pH 7.5 and 50 mM NaCl). Reactions were started by the addition of 1–2 pmol of transposase and incubated at 30°C for 4–16 h. Reactions were stopped by heating at 75°C for 10 min, and were analysed by agarose gel electrophoresis or extracted with phenol and ethanol precipitated before electroporation into the *recA*λ-lysogen strain DS964.

### Cleavage and integration assays

Oligonucleotides carrying ISY*100* terminal sequences were 5′ end-labelled with T4 polynucleotide kinase and [γ-^32^P]-ATP, and annealed to their unlabelled complementary strands. Approximately 0.1 pmol of labelled oligonucleotide and 1 μg of poly(dI-dC) were incubated with 0.65 pmol of purified transposase in 10 μl of transposase assay buffer at 30°C for 4 h, with or without the addition of 20% DMSO. Reactions were stopped by the addition of an equal volume of sequencing loading dye, and samples were run on 8% polyacrylamide sequencing gels as described above.

Integration assays were carried out the same way, in 20 μl of transposase assay buffer containing 0.1 pmol of labelled double-stranded oligonucleotides representing pre-cut transposon ends and 0.5 μg of pH2 target plasmid DNA. After incubation at 30°C for 4 h, reactions were stopped by heating at 75°C for 10 min and were analysed by electrophoresis on agarose gels run in TAE (40 mM Tris-acetate, 1 mM EDTA, pH 7.8) or denaturing (50 mM NaOH, 1 mM EDTA) running buffers.

### Mapping cleavage positions on supercoiled substrates

pISY*100*-kan and pXF106 were cleaved with transposase as described above. The vector fragment from pISY*100*-kan and the excised transposon fragment from pXF106 were gel-purified from agarose gels and treated for 15 min at 37°C with terminal deoxynucleotidyl transferase (Invitrogen) in the recommended buffer containing either 2 mM dCTP or dGTP. Vector products were subjected to four cycles of PCR with Pfu polymerase and primers poly G (5′-cccccatccatatgaagcttGGGGGGGGGGGG) and VecL (5′-TCAGGACGCGTCAGCGGGTGTTG) or VecR (5′-TGAGCATCGATTTTTGTGATGCTC), while ETF products were amplified with primers poly C (5′-gggggatccatatgaagcttCCCCCCCCCCCC) and ISY*100*L (5′-TCCTATGGCGACGCTCTACT) or ISY*100*R (5′-GCAGAAGGCTTTGAAGGATG) (see Fig. 7). Products were further amplified for 25 cycles using primers VecL, VecR, ISY*100*L or ISY*100*R and primers identical to the 5′ ends of poly G or poly C (nest-G, 5′-cccccatccatatgaagcttg; nestC, 5′-gggggatccatatgaagcttc) as appropriate. PCR products were gel purified and cloned into pCR2.1-TOPO using the topo-TA cloning kit (Invitrogen) and sequenced by MWG-biotech.

## Supplementary material

The following supplementary material is available for this article:

Fig. S1 Transposase with a C-terminal 6-His tag was overexpressed in E. coli and purified by spermine precipitation and metal ion affinity chromatography.

Fig. S2 ISY100 transposase contains two N-terminal helix-turn-helix DNA-binding motifs and a C-terminal DDE catalytic domain.

This material is available as part of the online article from: http://www.blackwell-synergy.com/doi/abs/10.1111/j.1365-2958.2007.05842.x (This link will take you to the article abstract).
