# Supplementary material for: In vitro transposition of ISY100, a bacterial insertion sequence belonging to the Tc1/mariner family
Source: Mol Microbiol. 2007 Sep;65(6):1432–43. doi: 10.1111/j.1365-2958.2007.05842.x (PMC2170065; doi:10.1111/j.1365-2958.2007.05842.x)
Supplement: Fig. S1 — Transposase with a C-terminal 6-His tag was overexpressed in E. coli and purified by spermine precipitation and metal ion affinity chromatography. [file mmi0065-1432-SD1.pdf]

IN VITRO TRANSPOSITION OF ISY100, A BACTERIAL INSERTION SEQUENCE BELONGING TO THE Tc1/MARINER FAMILY

Xiaofeng Feng and Sean D. Colloms*

Institute of Biomedical and Life Sciences,
Division of Molecular Genetics,
University of Glasgow,
Anderson College,
56 Dumbarton Rd,
Glasgow G11 6NU,
Scotland UK.

* Corresponding author

Supplementary material

Fig. S1
Fig. S2

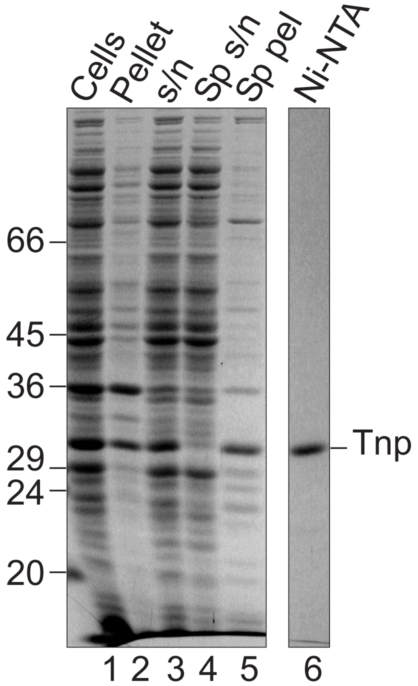

Fig. S1. Transposase with a C-terminal 6-His tag was over-expressed in *E. coli* and purified by spermine precipitation and metal ion affinity chromatography. Lane 1, whole cells; Lane 2, sonication pellet; Lane 3, sonication supernatant; Lane 4, spermine supernatant; Lane 5, spermine pellet; Lane 6, purified transposase eluted from Ni-NTA with 200 mM imidazole.

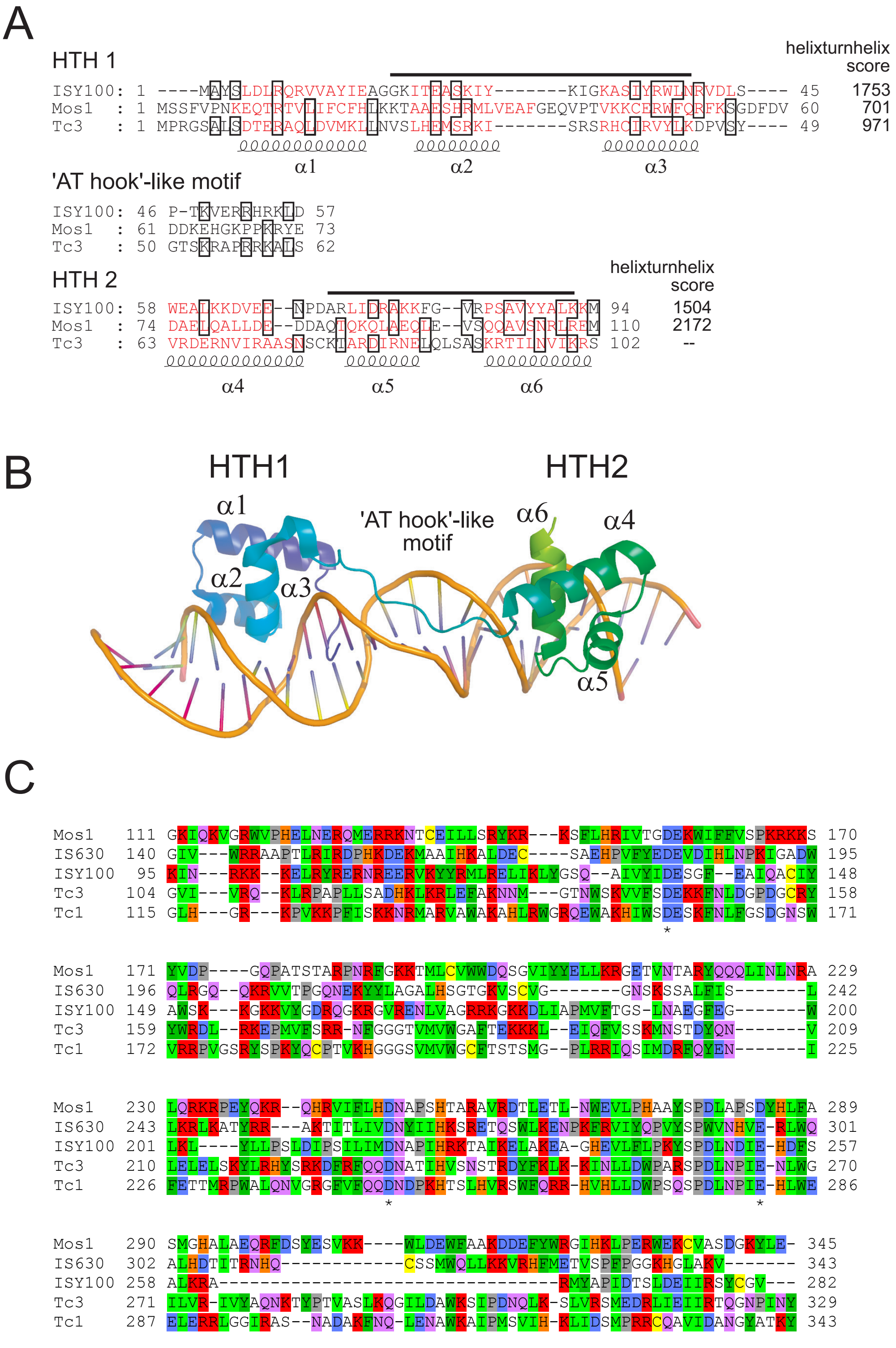

Fig. S2. ISY100 transposase contains two N-terminal helix-turn-helix DNA-binding motifs and a C-terminal DDE catalytic domain. (A) The sequence of the N-terminal 94 amino-acids of ISY100 transposase is shown aligned to the equivalent regions of *Mos1* mariner (gi: 157832) and Tc3 (gi: 464866) transposases. The alignment is based on matches between ISY100 and Mos1 transposases to the N-terminal domain of Tc3 transposase produced by the HHPRED server (Soding *et al*., 2005). Predicted (jpred) (Cuff and Barton, 2000) or actual (Tc3) α-helical regions are shown in red. Helix-turn-helix motifs predicted by the emboss program helixturnhelix (Dodd and Egan, 1990) are indicated by solid black bars over the sequence, and their scores are shown at the right. (B) Crystal stucture of the Tc3 N-terminal DNA-binding domain bound to DNA (pdb accession code: 1u78) (Watkins *et al*., 2004). Diagram produced using Pymol (DeLano, 2002) (C) MUSCLE Alignment of the C-terminal catalytic domains of ISY100, *Mos1*, Tc3, Tc1 (gi:15718656), and IS630 (gi:140943) transposases. Amino-acids are coloured according to the chemical nature of their side chains. The catalytic DDE(D) residues are indicated with asterisks (*). Muscle alignments were made using the MPI Bioinformatics toolkit (Biegert *et al*., 2006; Edgar, 2004).

References:

Biegert, A., Mayer, C., Remmert, M., Soding, J., and Lupas, A.N. (2006) The MPI Bioinformatics Toolkit for protein sequence analysis. *Nucleic Acids Res* **34:** W335-339.

Cuff, J.A., and Barton, G.J. (2000) Application of multiple sequence alignment profiles to improve protein secondary structure prediction. *Proteins* **40**: 502-511.

DeLano, W.L. (2002) The PyMOL Molecular Graphics System. *http://www.pymol.org*.

Dodd, I.B., and Egan, J.B. (1990) Improved detection of helix-turn-helix DNA-binding motifs in protein sequences. *Nucleic Acids Res* **18**: 5019-5026.

Edgar, R.C. (2004) MUSCLE: multiple sequence alignment with high accuracy and high throughput. *Nucleic Acids Res* **32**: 1792-1797.

Soding, J., Biegert, A., and Lupas, A.N. (2005) The HHpred interactive server for protein homology detection and structure prediction. *Nucleic Acids Res* **33**: W244-248.

Watkins, S., van Pouderoyen, G., and Sixma, T.K. (2004) Structural analysis of the bipartite DNA-binding domain of Tc3 transposase bound to transposon DNA. *Nucleic Acids Res* **32**: 4306-4312.
